# The validation of advanced-stage Hodgkin lymphoma international prognostic index (A-HIPI) in Turkish patients with classical Hodgkin lymphoma

**DOI:** 10.1007/s00277-025-06292-3

**Published:** 2025-03-12

**Authors:** Oguzhan Koca, Berk Ozyurt, Aysenur Umar, Deniz Ozmen, Tugrul Elverdi, Ayse Salihoglu, Muhlis Cem Ar, Zafer Baslar, Ahmet Emre Eskazan

**Affiliations:** 1 Department of Internal Medicine, Cerrahpasa Faculty of Medicine, Istanbul University-Cerrahpaşa, Istanbul, Turkey; 2 Cerrahpasa Faculty of Medicine, Istanbul University-Cerrahpasa, Istanbul, Turkey; 3 Division of Hematology, Department of Internal Medicine, Cerrahpasa Faculty of Medicine, Istanbul University-Cerrahpasa, Fatih, Istanbul, Turkey

**Keywords:** Clinical prediction models, IPS, A-HIPI, Prognostic score, Prognosis

## Abstract

In advanced-stage classical Hodgkin lymphoma (cHL), the prognosis has improved due to combination chemotherapy and PET/CT-guided treatment modification, resulting in a decreased prognostic capacity of IPS models. A novel model, A-HIPI, was found to be superior to IPS in predicting prognosis. In this study, we aimed to validate the A-HIPI model among Turkish cHL patients and compare its performance with other clinical prediction models. We retrospectively evaluated patients diagnosed with advanced-stage cHL between 2005 and 2018 at Istanbul University-Cerrahpaşa. We used IPS-7, IPS-3, and A-HIPI scores to calculate the C-index (Harrell’s Concordance Index) for discrimination; calibration intercept, and calibration slope for calibration. The models were compared using Akaike’s Information Criterion (AIC). Two hundred and seven patients were enrolled with a median follow-up of 75 months, 37 patients (17.9%) died. The 5-year PFS and OS were 66.6% and 84.9%, respectively. All three models were found to be prognostic for PFS and OS. The A-HIPI model was well-calibrated for PFS and OS in patients aged ≤65 years, but not calibrated for patients aged > 65 years. With A-HIPI, the respective C-index for PFS and OS was 0.605 and 0.740; whereas, for IPS-7 it was 0.598 and 0.684, and for IPS-3 it was 0.624 and 0.705. The lowest AIC value for OS was observed with the A-HIPI. The lowest AIC value for PFS was observed with IPS-3. This study validated the A-HIPI model in a homogeneous patient group for treatment protocol, with all follow-ups performed at a single center after the early 2000s in Turkey. The A-HIPI model demonstrated better performance than other models, except for patients aged > 65 years. A new clinical prediction model is needed for patients > 65 years, as IPS models are out of date and A-HIPI has not been validated for this group.

**Supplementary Information:**

The online version contains supplementary material available at 10.1007/s00277-025-06292-3.

## Introduction

Hodgkin lymphoma (HL) was first described in 1832 by Thomas Hodgkin, who reported seven patients with lymphadenopathy, splenomegaly, fever, night chills, and weight loss [1]. Many clinical prediction models have been developed to discriminate patients and provide the appropriate treatment since HL was defined.

Risk factor definitions are used for the appropriate treatment selection among risk groups in early-stage HL [2–4]. Meanwhile, International Prognostic Score (IPS) is used for advanced-stage HL. IPS was developed in 1998 using patient data from the 1980s [5]. Based on seven risk factors, it predicts overall survival (OS) and freedom from progression (FFP). With improvements in HL outcomes in the 21st century, there were concerns about the accuracy of the IPS model in predicting FFP and OS. However, in 2012, the model was validated in British Columbia, and it was found that IPS still holds prognostic value. The prognostic factors in the scoring were not changed, but the prediction of FFP and OS rates have been updated [6].

In another study conducted in 2015, out of the seven risk factors in IPS-7, age and stage were found to be significant for FFP, while age, stage, and hemoglobin level were significant for OS. Therefore, a simpler prognostic score system, IPS-3, was developed using three risk factors [7].

Despite the successes achieved in patient management, individuals with primary refractory disease and those who experience a recurrence after salvage therapy still have a poor prognosis [8]. The prognosis of cHL is generally inferior in elderly patients compared to younger individuals, with multiple factors contributing to worse outcomes, including higher rates of comorbidities and reduced treatment tolerance [9]. The development of prognostic indices is aimed at identifying these high-risk patients to provide more intensive treatment while also safeguarding patients with a good prognosis from unnecessary treatment toxicity.

New prognostic factors have been investigated as the power of IPS-7 and IPS-3 to discriminate between low-risk and high-risk group patients has decreased. Methods such as detecting tumor-associated macrophages with CD68 positivity [10–13], gene expression profiling from biopsy material [14], and checking EBV DNA expression at diagnosis and during follow-up [15] have been shown to be effective in predicting prognosis. However, they have not been widely adopted in clinical practice.

In late 2022, the Hodgkin Lymphoma International Study for Individual Care (HoLISTIC) consortium developed a new prognostic model called Advanced-Stage Hodgkin Lymphoma International Prognostic Index (A-HIPI). This new model was found to be more effective in predicting risk compared to other models [16]. In the A-HIPI model, age, gender, stage, bulky disease, absolute lymphocyte count, hemoglobin, and albumin levels were found to be prognostic. See Supplementary Table S1 for prognostic factors included in the IPS-7, IPS-3, and A-HIPI models.

IPS-7 and IPS-3 were developed using dichotomous data, such as age greater or less than 45. Since IPS-7 and IPS-3 were developed, clinical prediction modeling techniques have evolved. In contrast to IPSs, prognostic factors in A-HIPI are developed using continuous data.

This study aimed to validate and compare the A-HIPI model with other clinical prediction models in patients with advanced-stage cHL followed up at a single center in Turkey.

## Methods

The study was approved by the Istanbul University-Cerrahpasa Clinical Research Ethics Committee (Approval No: 83045809-604.01.01-799928, dated 09/10/2023). The study was conducted in accordance with the Declaration of Helsinki. The data from patients diagnosed with advanced stage cHL (2B, 3, 4) between 2005 and 2018, and who were treated by PET-guided approach at the Istanbul University-Cerrahpaşa Hematology Clinic, over 18 years old, and had a follow-up period of at least two chemotherapy cycles were examined. Patients with the histological subtype of nodular lymphocyte predominant Hodgkin lymphoma, a follow-up period shorter than two chemotherapy cycles, and those from whom more than 50% of prognostic data at the time of diagnosis could not be obtained were not included in the study.

Lugano classification was used for staging. According to the Lugano classification and the Cotswolds modification of the Ann Arbor system, bulky disease is defined as a single nodal mass (not multiple small nodes) larger than 10 cm, or the mass is larger than 1/3 of the transthoracic diameter at the same level. All included stage 2B patients had at least one of the established risk factors: a large mediastinal mass (> 1/3 of the thoracic diameter) or extranodal disease.

Progression-free survival (PFS) was defined as the time from pathological diagnosis to the first progression event, including disease progression, relapse, primary refractory disease, or death. Primary refractory disease refers to the failure to achieve complete remission with first-line therapy, while progressive disease was defined as an increase in lesion size or the appearance of new lesions during or after treatment. Progression that occurred during treatment or within the first 3 months after the completion of treatment is considered progressive disease. Relapse is defined as the reoccurrence of a disease after a temporary remission. OS was defined as the time from pathological diagnosis to death of any cause. Patients who did not experience death, progression, relapse, or refractory disease during follow-up and were lost to follow-up while in remission were censored during survival analyses.

### Statistical analysis

The data were analyzed using the Statistical Package for the Social Science (SPSS) 25.0. The Pearson chi-square test and Fisher’s exact test are used to compare qualitative data. The Mann-Whitney U test and the Kruskal-Wallis test are used to compare non-normally distributed variables. Survival analysis is conducted using the Kaplan-Meier method, and group differences are analyzed using the log-rank test. The impact of prognostic factors on survival is assessed using univariate and multivariate Cox regression analyses. A p-value of less than 0.05 is considered statistically significant.

Patients with missing data exceeding 50% were excluded from the study. For cases with less missing data, the missing data was addressed using the multiple imputation method. This method was applied to resolve missing values of hemoglobin, leukocyte, lymphocyte, and albumin at the time of diagnosis (Table 1). A-HIPI scores were calculated using the model equation provided in the model’s development study [16].

**Table 1:** Patient characteristics

Characteristic	(*N* = 207)
**Median age**,** years (range)**	37 (18–82)
**Age**,** categorical**,** n (%)**	
18–65 years	188 (90.8)
> 65 years	19 (9.2)
**Sex**,** n (%)**	
Female	87 (42.0)
Male	120 (58.0)
**Histology**,** n (%)**	
Nodular sclerosis	100 (48.3)
Mixed cellularity	66 (31.9)
Lymphocyte rich	3 (1.4)
Lymphocyte depleted	7 (3.4)
Not otherwise specified	31 (15.0)
**Stage**,** n (%)**	
Stage 2B	58 (28.0)
Stage 3	77 (37.2)
Stage 4	72 (34.8)
**B symptoms**,** n (%)**	161 (77.8)
**Bulky disease**,** n (%)**	36 (17.4)
**Extranodal disease**,** n (%)**	71 (34.3)
**Large mediastinal mass**,** n (%)**	24 (13.5)
**Median follow-up**,** months (IQR)**	75 (39–116)
**Missing counts before multiple imputation**,** n (%)**	
Hemoglobin	12 (5.8)
Leukocyte	11 (5.3)
Lymphocyte	20 (9.7)
Albumin	31 (15.0)

The performance, calibration, and discriminatory ability of different clinical prediction models were assessed using discrimination and calibration analyses. Discrimination was evaluated using Harrell’s Concordance Index (C-index), which measures the model’s ability to differentiate between individuals at higher and lower risk of experiencing an event [17]. The C-index ranges from 0 to 1, with 1 indicating perfect discrimination.

Calibration analysis was performed using calibration plots, where the agreement between predicted and observed outcomes was assessed. A calibration curve closely following the reference (ideal) line indicates better calibration. Calibration slope and calibration intercept were used as additional metrics to quantify calibration quality, with a slope close to 1 and an intercept close to 0 suggesting optimal calibration [18]. RStudio software was used to create calibration plots.

Additionally, model fit and complexity were compared using the Akaike Information Criterion (AIC). AIC provides a relative measure of model quality by balancing goodness of fit against model complexity, with lower AIC values indicating better model performance while minimizing overfitting [19]. In this study, AIC values were compared to assess the relative performance of the different models.

## Results

Patient characteristics are summarized in Table 1. Median age was 37 years. 9.2% of the 207 patients are over the age of 65. The median follow-up period is 75 months. Of the patients, 28% had stage 2B disease, 37.2% had stage 3 disease, and 34.8% had stage 4 disease. In total, 71 patients (34.3%) had extranodal disease, and 24 patients (13.5%) had a large mediastinal mass. The A-HIPI model calculated the expected 5-year PFS and OS for 207 patients estimate to be 76.6% and 92.3%, respectively.

All but five patients (97.6%) had received ABVD regimen as first-line therapy. Details of the first- and second-line treatment protocols and the number of cycles administered can be found in Supplementary Tables S2 and S3. Primary refractory disease was observed in 35 patients (16.9%), relapse was observed in 35 patients (16.9%), and progressive disease occurred in 14 patients (6.8%). RT was applied to 49 patients (23.7%). Hematopoietic stem cell transplantation was performed in 35 patients (16.9%). During the median follow-up of 75 months, 37 patients died, and 74 experienced any PFS event. 2-year PFS and OS were 74.5% and 91.8%; 5-year PFS and OS were 66.6% and 84.9% (Supplementary Figures S1 and S2), respectively.

**Fig. 1:**
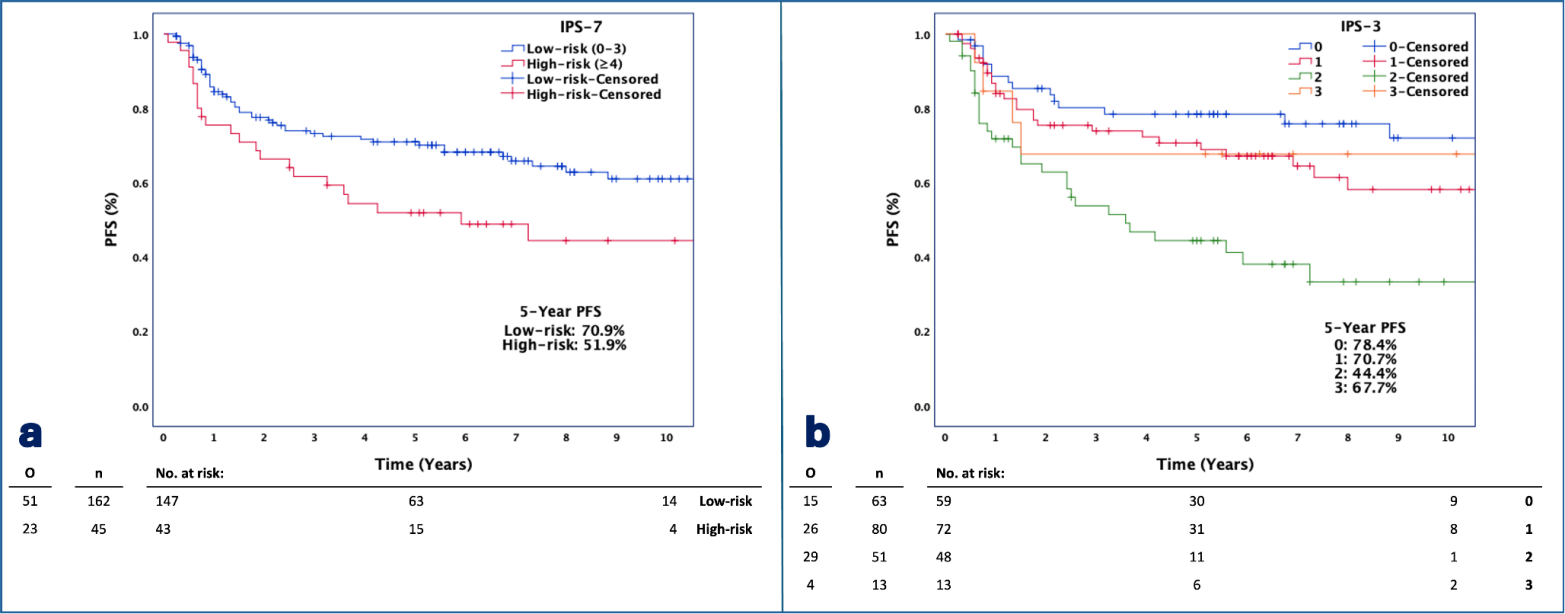
Kaplan-Meier Plots and number at risk tables of PFS stratified by IPS-7 (2 groups) (**a**) and IPS-3 (**b**) score

In Cox regression analyses, it was determined that PFS increased significantly as the A-HIPI PFS prediction increased (HR = 0.966, *p* = 0.012). When patients were divided into two groups according to IPS-7 scores, the risk of progression was found to be 1.73 times higher in the high-risk group (IPS-7: ≥4) than in the low-risk group (IPS-7: 0–3) (*p* = 0.029). When the reference group with an IPS-3 score of 0 was compared to the group with an IPS-3 score of 2, the risk of progression was found to be increased by 3.35 times (*p* < 0.001). When patients were categorized as low, intermediate, and high-risk based on the IPS-3 score, the risk of progression was 2.17 times higher in the intermediate-risk group (IPS-3: 1–2) compared to the low-risk group (IPS-3: 0) (*p* = 0.008) (Table 2). Kaplan-Meier plots of PFS stratified by the IPS-7 and IPS-3 scores are displayed in Fig. 1. 5-year PFS of the low-risk group is 70.9% and 51.9% for the high-risk group, according to IPS-7 (*p* = 0.026). 5-year PFS are 78.4%, 70.7%, 44.4%, and 67.7% for patient groups of IPS-3: 0, 1, 2, and 3 (*p* < 0.001).

**Table 2:** Univariate Cox regression analyzes for the effects of different factors on PFS [^a^Cox regression analyzes (CI, Confidence Interval (95%); HR, Hazard Ratio)]

	HR	CI	^a^*p* value
A-HIPI (*n* = 207)	0.966	0.939–0.992	**0.012**
IPS-7 (n, %)			
0–2 (100, 48.3%)	Reference		
3–4 (93, 45%)	1.963	1.209–3.185	**0.006**
≥ 5 (14, 6.7%)	1.459	0.562–3.791	0.438
IPS-7 (n, %)			
0–3 (162, 78.3%)	Reference		
≥ 4 (45, 21.7%)	1.734	1.060–2.837	**0.029**
IPS-3 (n, %)			
0 (63, 30.4%)	Reference		
1 (80, 38.6%)	1.566	0.829–2.958	0.167
2 (51, 24.6%)	3.350	1.790–6.269	**< 0.001**
3 (13, 6.3%)	1.443	0.479–4.348	0.515
IPS-3 (n, %)			
0 (63, 30.4%)	Reference		
1–2 (131, 63.3%)	2.169	1.224–3.844	**0.008**
3 (13, 6.3%)	1.441	0.478–4.342	0.517

**Fig. 2:**
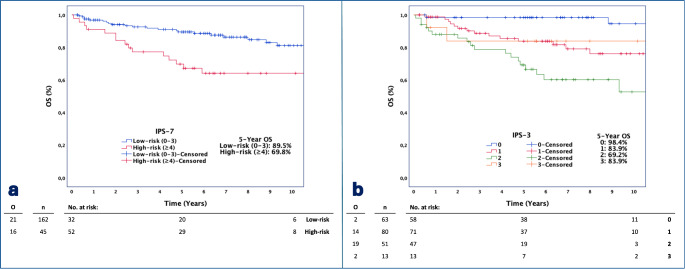
Kaplan-Meier Plots and number at risk tables of OS stratified by IPS-7 score (2 groups) (**a**) and IPS-3 (**b**) score

In Cox regression analyses, it was determined that OS increased significantly as the A-HIPI OS prediction increased (HR = 0.948, *p* < 0.001). When patients were divided into two groups according to IPS-7 scores, the risk of mortality was found to be 2.91 times higher in the high-risk group (IPS-7: ≥4) than in the low-risk group (IPS-7: 0–3) (*p* = 0.001). When the reference group with IPS-3 score of 0 was compared to the other groups, the risk of mortality was found to be increased by 6.64 times (*p* = 0.012) in the group with IPS-3 score of 1 and increased by 16.2 times (*p* < 0.001) in the group with IPS-3 score of 2. When patients were categorized as low, intermediate, and high-risk based on the IPS-3 score, the risk of mortality was 10.01 times higher in the intermediate-risk group (IPS-3: 1–2) compared to the low-risk group (IPS-3: 0) (*p* = 0.002) (Table 3). Kaplan-Meier plots of OS stratified by the IPS-7 and IPS-3 scores are displayed in Fig. 2. 5-year OS of the low-risk group is 89.5% and 69.8% for the high-risk group, according to IPS-7 (*p* = 0.001). 5-year OS are 98.4%, 83.9%, 69.2%, and 83.9% for patient groups of IPS-3: 0, 1, 2, and 3 (*p* < 0.001).

**Table 3:** Univariate Cox regression analyzes for the effects of different factors on OS [^a^Cox regression analyzes (CI, Confidence Interval (95%); HR, Hazard Ratio)]

	HR	CI	^a^*p* value	
A-HIPI (*n* = 207)	0.948	0.925–0.972	**< 0.001**	
IPS-7 (n, %)				
0–2 (100, 48.3%)	Reference			
3–4 (93, 45%)	4.164	1.884–9.203	**< 0.001**	
≥ 5 (14, 6.7%)	2.944	0.779–11.131	0.111	
IPS-7 (n, %)				
0–3 (162, 78.3%)	Reference			
≥ 4 (45, 21.7%)	2.905	1.515–5.572	**0.001**	
IPS-3 (n, %)				
0 (63, 30.4%)	Reference			
1 (80, 38.6%)	6.635	1.507–29.224	**0.012**	
2 (51, 24.6%)	16.204	3.763–69.768	**< 0.001**	
3 (13, 6.3%)	5.962	0.837–42.471	0.075	
IPS-3 (n, %)				
0 (63, 30.4%)	Reference			
1–2 (131, 63.3%)	10.010	2.399–41.773	**0.002**	
3 (13, 6.3%)	5.925	0.832–42.193	0.076	

**Fig. 3:**
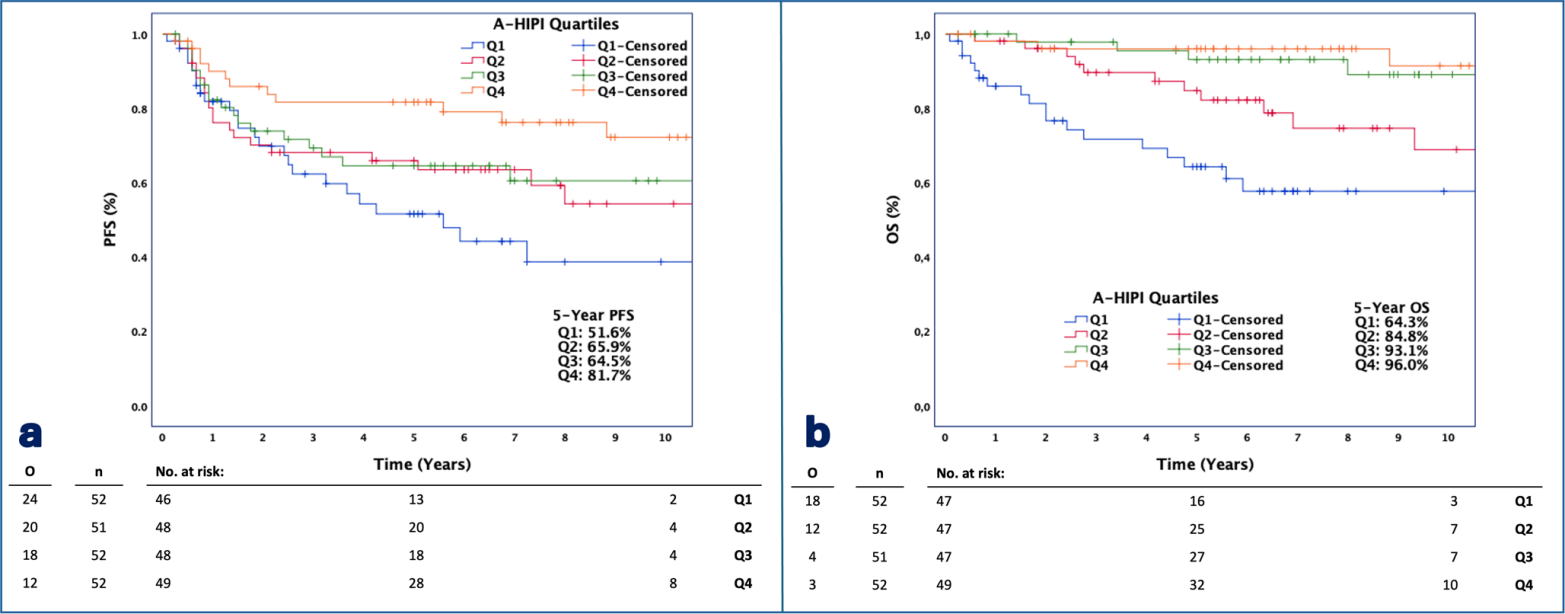
Kaplan-Meier Plots and number at risk tables of PFS (**a**) and OS (**b**) stratified by A-HIPI quartiles

Further, all patients were categorized into quartiles (Q1, Q2, Q3, Q4) according to A-HIPI scores and evaluated with Kaplan-Meier analyses for survival. Q1 represents the group with the lowest expected/predicted survival, while Q4 represents the group with the highest expected/predicted survival. 5-year PFS rates in quartiles (Q1-Q4) were 51.6%, 65.9%, 64.5%, 81.7% respectively (*p* = 0.030) and 5-year OS rates are 64.3%, 84.8%, 93.1%, 96.0% respectively (*p* < 0.001). Kaplan-Meier Plots of PFS and OS stratified by A-HIPI score are given in Fig. 3.

Additionally, patients were divided into subgroups as ≤ 65 years of age (*n* = 188) and > 65 years of age (*n* = 19). 5-year PFS and OS were 68.8% and 87.1% for patients ≤ 65 years of age and 39.5% and 59% for patients > 65 years of age. In Cox regression analyses in patients > 65 years of age, no statistically significant relationship was found between A-HIPI, IPS-7, IPS-3 scores and survival.

**Fig. 4:**
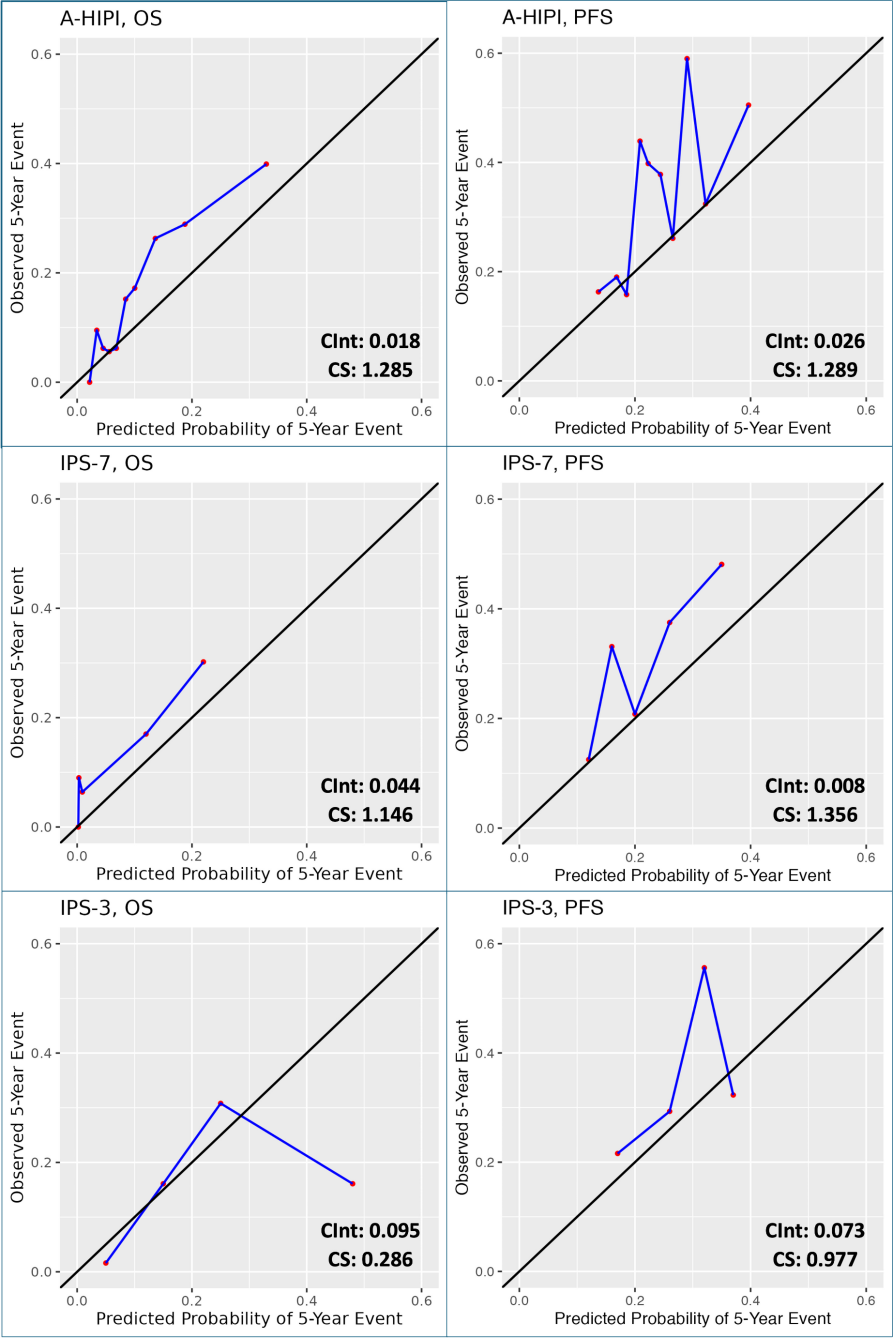
Calibration plots for A-HIPI, IPS-7 and IPS-3 in the entire sample (CInt, Calibration Intercept; CS, Calibration Slope)

**Fig. 5:**
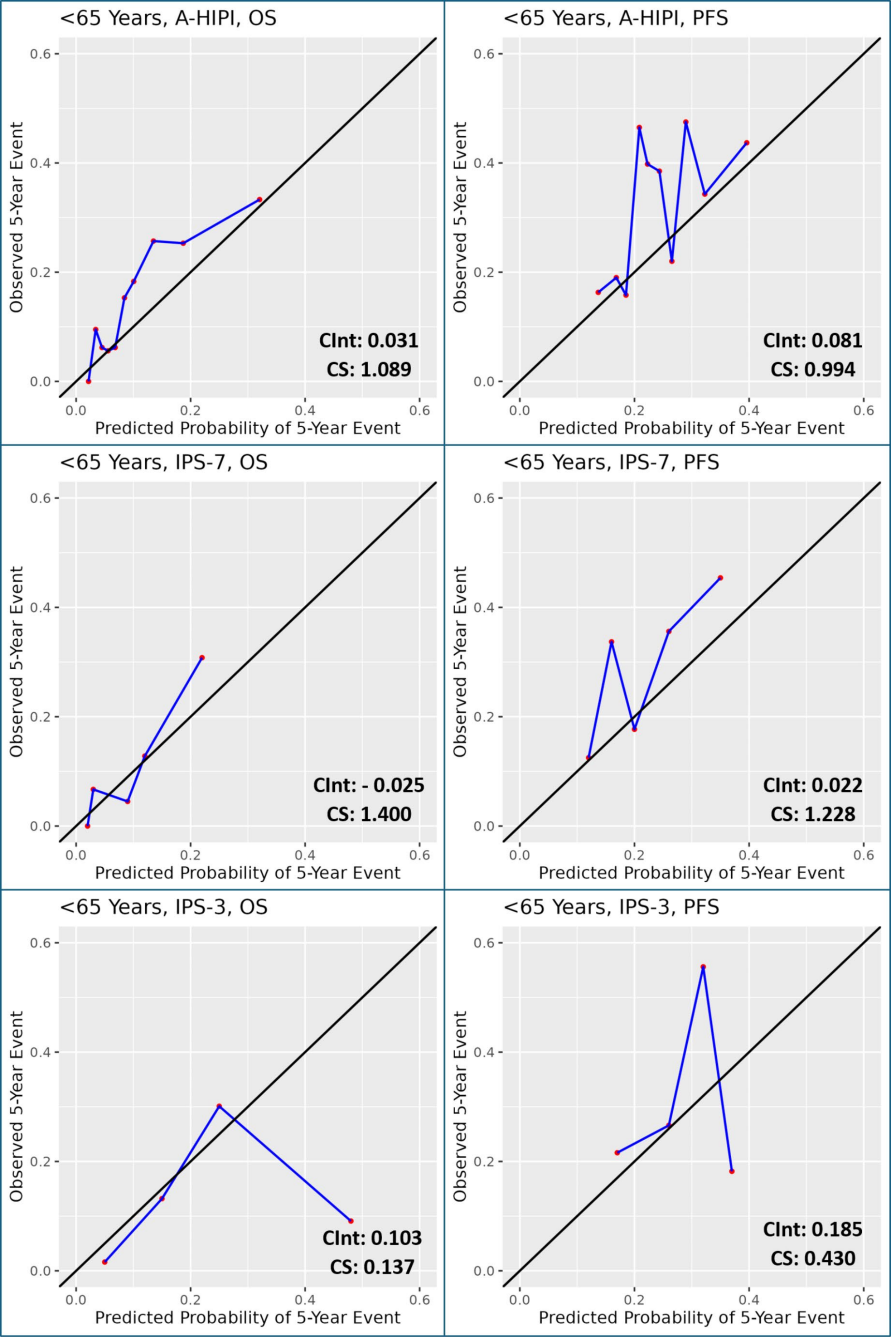
Calibration plots for A-HIPI, IPS-7 and IPS-3 in patients ≤65 years of age (CInt, Calibration Intercept; CS, Calibration Slope)

Calibration plots were created for each model to assess the calibration of clinical prediction models. Observed and predicted probabilities of 5-year survival were compared to assess calibration. For the A-HIPI model, patients were divided into deciles based on their predicted event risk. For the IPS-7 and IPS-3 models, patients were categorized based on their IPS scores (for IPS-7: 0, 1, 2, 3 and ≥ 4; for IPS-3: 0, 1, 2, 3). Calibration plots for PFS and OS are given in Figs. 4 and 5. Since 19 patients over the age of 65 were in the sample, calibration intercepts and slopes were not calculated separately for this group.

The A-HIPI model appears well calibrated for OS and PFS in the entire sample and in patients ≤ 65 years of age but slightly underestimates the probability of event in the high-risk group (Figs. 4 and 5). For the A-HIPI OS model, calibration intercept and calibration slope were 0.018 and 1.285; for the A-HIPI PFS model, calibration intercept and calibration slope were 0.026 and 1.289. For the A-HIPI OS model in patients ≤ 65 years of age, calibration intercept and calibration slope were 0.031 and 1.089; for the A-HIPI PFS model in patients ≤ 65 years of age, calibration intercept and calibration slope were 0.081 and 0.994. It was observed that the calibration slope of the A-HIPI model approached 1 in the calibration plots created in patients aged ≤ 65 years, and visually, the calibration of the model was observed to be better in patients aged ≤ 65 years (Figs. 4 and 5).

Discrimination analyses with Harrel’s Concordance Index in the entire sample, in patients ≤ 65 years of age and patients > 65 years of age, are given in Supplementary Table S4.

For OS, the AIC values of IPS-7, IPS-3, and A-HIPI were determined to be 2205.19, 2202.05, and 2192.37. For PFS, the AIC values of IPS-7, IPS-3, and A-HIPI were determined to be 2214.76, 2209.78, and 2211.12. The A-HIPI model has the lowest AIC value for OS (A-HIPI, IPS-7 ΔAIC: 12.8; A-HIPI, IPS-3 ΔAIC: 9.7). The IPS-3 model has the lowest AIC value for PFS (IPS-3, A-HIPI ΔAIC: 1.3; IPS-3, IPS-7 ΔAIC: 5.0) (Supplementary Table S5).

## Discussion

As treatment modalities and disease outcomes in HL change, it has been argued in the 21st century that clinical prediction models are ineffective in new patient populations, and new prediction models are required [6, 7, 10–16]. The historical IPS model, initially published by Hasenclever et al. in 1998, was revised in 2012 [5, 6]. The IPS-3 score was introduced in 2015, and with the advancement of statistical analysis methods, the A-HIPI model, which allows for the use of continuous data, was published in 2023 [5–7, 16]. In this study, we compared different clinical prediction models for advanced-stage cHL patients regarding calibration and discrimination.

The median age was 37 years, which was higher compared to the updated IPS-7, IPS-3, and A-HIPI studies (median values of the age at diagnosis were 32, 33, and 33, respectively) [6, 7, 16]. In our study, patients over 65 were included, consistent with the updated IPS-7 and IPS-3 studies, whereas the original IPS-7 and A-HIPI studies excluded them.

MScHL and LDcHL histological subtypes were more represented in our study than in studies developing clinical prediction models (Supplementary Table S6). The MScHL subtype, which is more associated with socioeconomic level and EBV infection, constituted 31.9% of the patients and the LDcHL subtype was found in 3.4% in our study. The rates of NScHL, the most common histological subtype, were 63%, 78%, 72%, and 74.2% in previous studies and 48.3% in our study. The higher incidence of MScHL and LDcHL in Turkey compared to Europe and America is likely due to the relationship between cHL histological subtype distribution and factors such as geography, race, socioeconomic status, and EBV [20]. Geographic differences in cHL epidemiology and treatment outcomes highlight the need for region-specific validation of clinical prediction models, as studies from non-Western countries suggest that socio-economic and healthcare system differences may significantly impact survival [21].

In the current study and the A-HIPI study, stage 2 patients were underrepresented compared to the updated IPS-7 and IPS-3 studies. In the last two decades, diagnostic and staging methods, especially PET/CT, have developed, staging has become more precise and the number of stage 2 patients receiving advanced disease treatment has decreased today, which may be a factor here [6].

The patients in our study have a higher baseline risk of death and progression. In our study, 51.7% of the patients had an IPS-7 score of 3 or above, while in previous studies (original IPS-7, updated IPS-7, IPS-3, and A-HIPI), the percentages were 42%, 40%, 31%, and 37.4%, respectively. This may explain why the 5-year OS and PFS rates are lower in the present study compared to the literature. Furthermore, it is essential to consider the impact of socioeconomic factors in our country, which are not included in clinical prediction models and whose effect on disease outcome has been shown, is different from the North American and European populations where models were developed, is thought to be a factor in the lower survival rates [22]. However, the survival rates in our study are similar to those in another study conducted in Brazil in a similar sample size. In our study, 5-year PFS and OS were found to be 66.6% and 84.9%, and in the study conducted in Brazil, 5-year PFS and OS were found to be 68.4% and 86.0% [23]. This suggests the need to validate these models in geographies with different survival rates than North America and Europe, where clinical prediction models are developed, since histological subtype distribution, socioeconomic level, and EBV infection prevalence differ depending on the geography.

In our study, all but five patients received the ABVD regimen. This created a homogeneous patient group because those patients did not receive chemotherapy that does not contain doxorubicin and has lower effectiveness against ABVD, such as MOPP. Diagnosis and follow-up of all patients occurred in the 21st century (after 2005), unlike the studies where the models were developed, and all models were still found to be prognostic. Supplementary Table S6 was created to compare the patient characteristics provided in the current study with those in studies where models were developed.

When the patients were divided into four groups based on the IPS-3 score, we observed a decrease in survival as the IPS-3 score went from 0 to 2 in the Kaplan-Meier survival plots. However, the survival plot for the subgroup with IPS-3: 3 appeared incompatible. This could be due to the small sample size of just 13 (6.3%) patients in this subgroup (Figs. 1B and 2B).

In the original IPS-7 and A-HIPI studies, patients aged over 65 were omitted. However, IPS-7 was later validated for this age group with an update in 2012 [5, 6, 16]. In our study, no significant relationship was found with any model in the analysis of OS and PFS among patients over 65 years of age (*n* = 19). It is important to note that a limitation of our study is the insufficient sample size to validate clinical prediction models for this age group. In a study evaluating the performance of the A-HIPI model, using the Danish National Lymphoma registry system, patients diagnosed with advanced-stage HL were divided into two groups, under 65 years of age and over 65 years of age. However, the model showed poor performance in patients over 65 years of age [24]. Therefore, the A-HIPI model is not currently suitable for use in patients over 65 years of age. It is essential to either update the existing model or develop a new clinical prediction model tailored to this age group.

There were no calibration analyses in the development and validation studies of IPS-7 and IPS-3 [5–7]. However, the A-HIPI study observed that the model is well-calibrated through calibration analysis [16]. In our study, the A-HIPI model appears well-calibrated in the entire sample and the group aged 65 and under. However, upon visually evaluating the calibration plots and considering the proximity to the value of 1 in the calibration slope, the A-HIPI model is better calibrated in the 65 and underage group. Our study’s findings support existing literature, indicating that the A-HIPI model is not appropriate for use in patients over 65 years of age [24]. Additionally, in our study, the A-HIPI model slightly underestimated the probability of developing events only in high-risk patients.

Based on the visual examination of calibration plots, calibration intercepts, and calibration slopes, the A-HIPI model shows the best calibration among the three models for both PFS and OS. In our study, it seems that the IPS-3 model is not properly calibrated for predicting survival. This is because very few patients have an IPS-3 score of 3, and the expected and observed event rates are different in that subgroup, showing a significant deviation at the high end of the calibration plot. When visually examining the calibration plot for the IPS-3 model, it seems to effectively predict the risk for patients in the low and medium-risk groups (IPS-3: 0–2).

The calibration analyses for OS and PFS are consistent with the findings in the literature [16, 23, 24]. In the development study, the A-HIPI model showed better calibration than IPSs for OS and PFS [16]. Validation studies in Denmark and Brazil confirmed that the A-HIPI model was well-calibrated for OS and PFS, with the Danish study reporting a calibration slope of 0.99 for OS; however, neither study compared A-HIPI with other models [23, 24].

In discrimination analyses showing the power of clinical prediction models to distinguish patients with good prognosis and poor prognosis, the model with the highest C-index for OS in the entire sample and patients aged ≤ 65 years is A-HIPI. For PFS, the model with the highest C-index is IPS-3. For PFS, the A-HIPI model has a lower C-index than IPS-3 but a higher C-index than IPS-7. (Supplementary Table S4). Discrimination analyses in our study are consistent with the development and validation studies of A-HIPI [16, 25]. The original A-HIPI study reported better discrimination for OS but worse discrimination for PFS [16]. Similarly, an Italian validation study found better discrimination for OS, but worse for PFS compared to IPS-7 [25]. In contrast, a Danish study showed A-HIPI outperformed IPS-7 in discriminating both OS and PFS [24]. A Brazilian validation study reported C-index values of 0.60 for PFS and 0.69 for OS, though without comparison to IPS models [23].

Based on the AIC analysis, the A-HIPI model was more compatible with our study data for determining prognosis in terms of OS, while the IPS-3 model was more compatible in terms of PFS. The results of the AIC analysis for selecting the appropriate prediction model in our study are similar to those of the study conducted in Italy [25]. The Italian study compared the A-HIPI model with the IPS-7 model. The AIC value showed that the A-HIPI model was more suitable for OS, and less suitable for PFS [25]. AIC analysis was not performed in other validation studies of the A-HIPI model [23, 24].

Recent evidence from a comprehensive analysis of randomized trials conducted by the German Hodgkin Study Group (GHSG) demonstrated a strong correlation between PFS and OS (Pearson r: 0.72–0.83), suggesting that PFS can serve as a reliable surrogate for OS in cHL clinical trials [26]. Given these findings, the relative performance of A-HIPI in predicting PFS warrants further discussion. In our study, A-HIPI showed the best discrimination for OS, but had lower predictive power for PFS compared to IPS-3. The lower AIC value of IPS-3 for PFS suggests that a simpler model may be preferred when focusing on this endpoint. However, as PFS and OS are strongly correlated, a model excelling in OS prediction may still be valuable for long-term risk stratification. Further studies are needed to refine prognostic models that optimally predict both PFS and OS in diverse patient populations. Additionally, the relatively small difference between A-HIPI and other models in our study is likely due to the limited sample size, which represents a limitation of our study.

Considering the original study of the A-HIPI, the validation study conducted in Italy, and our study, the A-HIPI model demonstrates good performance in predicting prognosis, seems to be superior to other models in terms of calibration, superior in discriminating OS, but shows slightly lower performance in discriminating PFS [16, 25]. This suggests that different prognostic factors may affect 5-year treatment failure and are not included in the models. In the original A-HIPI study, it was thought that patients’ baseline risk scores at the time of diagnosis have become less important in predicting PFS due to the interim PET/CT-guided treatment approach. This has been thought to be one of the factors that cause the A-HIPI model to have discrimination results for PFS similar to IPS models [16].

A-HIPI’s ability to stratify patients based on OS suggests its potential utility in guiding treatment intensity decisions. For high-risk patients, intensification strategies such as BV-AVD or eBEACOPP could be considered over ABVD to improve outcomes. Conversely, for low-risk patients, de-escalation approaches should be explored to minimize toxicity while maintaining efficacy. However, since A-HIPI demonstrated lower discrimination for PFS, clinicians should exercise caution when using it to predict short-term treatment failure.

In conclusion, clinical prediction models can be used in large populations by validating them in cohorts with different characteristics. Once developed, the A-HIPI model was validated in Brazil, Denmark, and Italy [23–25]. Following these studies, our study validated the A-HIPI model in a group of patients diagnosed with advanced-stage cHL who were followed up from a single tertiary center in Turkey. After conducting calibration and discrimination analyses, the A-HIPI model demonstrated the best performance among these three models when patients over 65 years of age were excluded. A new clinical prediction model is needed for patients over 65 years of age, as IPS models are out of date and the A-HIPI model is not validated in this group. In addition, further studies are needed to include dynamic factors in the follow-up and treatment process that are not included in clinical prediction models, such as chemotherapeutic selection and interim PET/CT-guided treatment modification, in addition to the baseline risk factors detected at the time of diagnosis with larger patient cohorts.

## Electronic supplementary material

Below is the link to the electronic supplementary material.

Supplementary Material 1

## Data Availability

No datasets were generated or analysed during the current study.

## References

[1] Hodgkin (1832) On some morbid appearances of the absorbent glands and spleen. J R Soc Med MCT–17:68–114. 10.1177/095952873201700106 10.1177/095952873201700106 PMC2116706 20895597

[2] Cheson BD, Fisher RI, Barrington SF et al (2014) Recommendations for initial evaluation, staging, and response assessment of hodgkin and Non-Hodgkin lymphoma: the Lugano classification. J Clin Oncol 32:3059–3067. 10.1200/JCO.2013.54.8800 25113753 10.1200/JCO.2013.54.8800 PMC4979083

[3] Engert A, Plütschow A, Eich HT et al (2010) Reduced treatment intensity in patients with Early-Stage Hodgkin’s lymphoma. N Engl J Med 363:640–652. 10.1056/NEJMoa1000067 20818855 10.1056/NEJMoa1000067

[4] Klimm B, Goergen H, Fuchs M et al (2013) Impact of risk factors on outcomes in early-stage Hodgkin’s lymphoma: an analysis of international staging definitions. Ann Oncol 24:3070–3076. 10.1093/annonc/mdt413 24148816 10.1093/annonc/mdt413

[5] Hasenclever D, Diehl V, Armitage JO et al (1998) A prognostic score for advanced Hodgkin’s disease. N Engl J Med 339:1506–1514. 10.1056/NEJM199811193392104 9819449 10.1056/NEJM199811193392104

[6] Moccia AA, Donaldson J, Chhanabhai M et al (2012) International prognostic score in Advanced-Stage Hodgkin’s lymphoma: altered utility in the modern era. J Clin Oncol 30:3383–3388. 10.1200/JCO.2011.41.0910 22869887 10.1200/JCO.2011.41.0910

[7] Diefenbach CS, Li H, Hong F et al (2015) Evaluation of the international prognostic score (IPS-7) and a simpler prognostic score (IPS-3) for advanced hodgkin lymphoma in the modern era. Br J Haematol 171:530–538. 10.1111/bjh.13634 26343802 10.1111/bjh.13634 PMC4881845

[8] Arai S, Fanale M, deVos S et al (2013) Defining a hodgkin lymphoma population for novel therapeutics after relapse from autologous hematopoietic cell transplant. Leuk Lymphoma 54:2531–2533. 10.3109/10428194.2013.798868 23617324 10.3109/10428194.2013.798868

[9] Çokgezer S, Elverdi T, Salihoğlu A et al (2022) Treatment responses, toxicity, and survival in patients with classical hodgkin lymphoma aged ≥ 50 years: A Single-Center experience over two decades. Cancer Manag Res 14:1911–1921. 10.2147/CMAR.S363235 35698602 10.2147/CMAR.S363235 PMC9188373

[10] Steidl C, Lee T, Shah SP et al (2010) Tumor-Associated macrophages and survival in classic Hodgkin’s lymphoma. N Engl J Med 362:875–885. 10.1056/NEJMoa0905680 20220182 10.1056/NEJMoa0905680 PMC2897174

[11] Kamper P, Bendix K, Hamilton-Dutoit S et al (2011) Tumor-infiltrating macrophages correlate with adverse prognosis and Epstein-Barr virus status in classical Hodgkin’s lymphoma. Haematologica 96:269–276. 10.3324/haematol.2010.031542 21071500 10.3324/haematol.2010.031542 PMC3031695

[12] Tan KL, Scott DW, Hong F et al (2012) Tumor-associated macrophages predict inferior outcomes in classic hodgkin lymphoma: a correlative study from the E2496 intergroup trial. Blood 120:3280–3287. 10.1182/blood-2012-04-421057 22948049 10.1182/blood-2012-04-421057 PMC3476539

[13] Touati M, Delage-Corre M, Monteil J et al (2015) CD68-positive tumor-associated macrophages predict unfavorable treatment outcomes in classical hodgkin lymphoma in correlation with interim fluorodeoxyglucose-positron emission tomography assessment. Leuk Lymphoma 56:332–341. 10.3109/10428194.2014.917636 24766492 10.3109/10428194.2014.917636

[14] Scott DW, Chan FC, Hong F et al (2013) Gene Expression–Based model using Formalin-Fixed Paraffin-Embedded biopsies predicts overall survival in Advanced-Stage classical hodgkin lymphoma. J Clin Oncol 31:692–700. 10.1200/JCO.2012.43.4589 23182984 10.1200/JCO.2012.43.4589 PMC3574267

[15] Kanakry JA, Li H, Gellert LL et al (2013) Plasma Epstein-Barr virus DNA predicts outcome in advanced hodgkin lymphoma: correlative analysis from a large North American cooperative group trial. Blood 121:3547–3553. 10.1182/blood-2012-09-454694 23386127 10.1182/blood-2012-09-454694 PMC3643756

[16] Rodday AM, Parsons SK, Upshaw JN et al (2023) The Advanced-Stage hodgkin lymphoma international prognostic index: development and validation of a clinical prediction model from the holistic consortium. J Clin Oncol 41:2076–2086. 10.1200/JCO.22.02473 36495588 10.1200/JCO.22.02473 PMC10082254

[17] Alba AC, Agoritsas T, Walsh M et al (2017) Discrimination and calibration of clinical prediction models. JAMA 318:1377. 10.1001/jama.2017.12126 29049590 10.1001/jama.2017.12126

[18] Van Calster B, McLernon DJ, van Smeden M et al (2019) Calibration: the Achilles heel of predictive analytics. BMC Med 17:230. 10.1186/s12916-019-1466-7 31842878 10.1186/s12916-019-1466-7 PMC6912996

[19] Burnham KP, Anderson DR (2002) Model selection and multimodel inference: A practical Information-Theoretic approach. Springer Nature

[20] Weinreb M, Day PJ, Niggli F et al (1996) The role of Epstein-Barr virus in Hodgkin’s disease from different geographical areas. Arch Dis Child 74:27–31. 10.1136/adc.74.1.27 8660041 10.1136/adc.74.1.27 PMC1511586

[21] Brittain D, Akhtar S, Rodrigues S et al (2024) Treatment patterns and clinical outcomes in patients with hodgkin lymphoma from Saudi Arabia, Türkiye, and South Africa: subgroup analysis from the international multicenter retrospective B-HOLISTIC study. Turk J Haematol 41:211–224. 10.4274/tjh.galenos.2024.2024.0181 39463021 10.4274/tjh.galenos.2024.2024.0181 PMC11628755

[22] Biasoli I, Castro N, Delamain M et al (2018) Lower socioeconomic status is independently associated with shorter survival in hodgkin lymphoma patients—An analysis from the Brazilian hodgkin lymphoma registry. Int J Cancer 142:883–890. 10.1002/ijc.31096 29023692 10.1002/ijc.31096

[23] Biasoli I, Buccheri V, Moreira FR et al (2023) External validation of the Advanced-Stage hodgkin lymphoma international prognostic index (A-HIPI): strong calibration in the Brazilian prospective hodgkin lymphoma registry. Blood 142:6200. 10.1182/BLOOD-2023-173318

[24] Jørgensen RK, Eloranta R, Christensen S JH, et al (2023) Age-Based validation of the Advanced-Stage hodgkin lymphoma international prognostic index (A-HIPI) in a Real-World Danish study: suboptimal performance in older patients. Blood 142:4455. 10.1182/blood-2023-178498

[25] Cellini A, Adele Cavarretta C, Angotzi F et al (2023) P1075: ADVANCED-STAGE CHL INTERNATIONAL PROGNOSTICATION INDEX: EXTERNAL VALIDATION IN A SINGLE-CENTER RETROSPECTIVE PATIENT DATASET AND COMPARISON TO THE HASENCLEVER IPS. Hemasphere 7

[26] Bröckelmann PJ, Müller H, Fuchs M et al (2024) Correlation between progression-free and overall survival in patients with hodgkin lymphoma: a comprehensive analysis of individual patient data from randomized German hodgkin study group (GHSG) trials. 10.1016/j.annonc.2024.12.009. Annals of Oncology 10.1016/j.annonc.2024.12.009 39706337

