# Supplementary material for: The validation of advanced-stage Hodgkin lymphoma international prognostic index (A-HIPI) in Turkish patients with classical Hodgkin lymphoma
Source: Ann Hematol. 2025 Mar 12;104(3):1765–75. doi: 10.1007/s00277-025-06292-3 (PMC12031855; doi:10.1007/s00277-025-06292-3)

**Supplementary Table S1.** Prognostic factors included in the IPS-7, IPS-3, and A-HIPI models.

| **IPS-7** | **IPS-3** | **A-HIPI** |
| --- | --- | --- |
| Age | Age | Age |
| Hemoglobin | Hemoglobin | Hemoglobin |
| Stage 4 disease | Stage 4 disease | Stage |
| Albumin |  | Albumin |
| Male sex |  | Female sex |
| Leukocyte count |  | Bulky disease |
| Lymphocyte count or ratio |  | Lymphocyte count |

**Supplementary Table S2.** First-line chemotherapy regimens and cycles administered.

| **Regimen** | **n** | **%** | **Cycles Received (Median)** |
| --- | --- | --- | --- |
| ABVD | 202 | 97.5 | 6 |
| AVD+BV | 1 | 0.5 | 6 |
| AVD | 1 | 0.5 | 4 |
| BEACOPP | 1 | 0.5 | 8 |
| CHOP | 2 | 1 | 7 |
| **TOTAL** | 207 | 100 | 6 |

**Supplementary Table S3.** Second-line chemotherapy regimens and cycles administered.

| **Regimen** | **n** | **%** | **Cycles Received (Median)** |
| --- | --- | --- | --- |
| ICE | 38 | 67.9 | 3 |
| BEACOPP | 6 | 10.7 | 5 |
| MOPP | 3 | 5.4 | 3.5 |
| DHAP | 2 | 3.6 | 4 |
| BV | 2 | 3.6 | 3 |
| ABVD | 1 | 1.8 | 4 |
| BENDAMUSTIN | 1 | 1.8 | 2 |
| ESHAP | 1 | 1.8 | 2 |
| GVP | 1 | 1.8 | 3 |
| CHLVPP | 1 | 1.8 | 5 |
| **TOTAL** | 56 | 100 | 3 |

**Supplementary Table S4.** Comparison of C-index for IPS-7, IPS-3, and A-HIPI, (PFS, progression-free survival; OS, overall survival).

|  | **PFS** | | **OS** | |
| --- | --- | --- | --- | --- |
|  | **C-index** | **CI** | **C-index** | **CI** |
| **Entire Sample** |  |  |  |  |
| IPS-7 | 0.598 | 0.513-0.683 | 0.684 | 0.580-0.788 |
| IPS-3 | 0.624 | 0.541-0.706 | 0.705 | 0.617-0.793 |
| A-HIPI | 0.605 | 0.525-0.685 | 0.740 | 0.642-0.838 |
| **≤65 years** |  |  |  |  |
| IPS-7 | 0.592 | 0.500-0.683 | 0.720 | 0.608-0.832 |
| IPS-3 | 0.614 | 0.525-0.703 | 0.716 | 0.620-0.812 |
| A-HIPI | 0.591 | 0.506-0.677 | 0.726 | 0.613-0.840 |
| **>65 years** |  |  |  |  |
| IPS-7 | 0.633 | 0.377-0.889 | 0.500 | 0.226-0.774 |
| IPS-3 | 0.622 | 0.362-0.883 | 0.538 | 0.246-0.831 |
| A-HIPI | 0.544 | 0.275-0.814 | 0.590 | 0.331-0.849 |

**Supplementary Table S5.** Comparison of AIC for IPS-7, IPS-3, and A-HIPI (AIC, Akaike's Information Criterion; PFS, progression-free survival; OS, overall survival).

|  | **PFS** | | **OS** | |
| --- | --- | --- | --- | --- |
|  | **AIC** | **ΔAIC (vs IPS-3)** | **AIC** | **ΔAIC (vs A-HIPI)** |
| IPS-7 | 2214.76 | 5.0 | 2205.19 | 12.8 |
| IPS-3 | 2209.78 | 0 | 2202.05 | 9.7 |
| A-HIPI | 2211.12 | 1.3 | 2192.37 | 0 |

**Supplementary Table S6.** Patient characteristics in the present study and reference studies (HSCT, Hematopoietic stem cell transplantation; NA, not available; NOS, not otherwise specified).

|  | **Original IPS-7** | | **Updated IPS-7** | | **IPS-3** | | **A-HIPI** | | **Current Study** | |
| --- | --- | --- | --- | --- | --- | --- | --- | --- | --- | --- |
|  | **N** | **%** | **N** | **%** | **N** | **%** | **N** | **%** | **N** | **%** |
| **Total** | 4695 |  | 740 |  | 854 |  | 4022 |  | 207 |  |
| **Stage** |  | | | | | | | | | |
| **Stage 1-2** | 603 | 13 | 308 | 42 | 314 | 37 | 1106 | 27.5 | 58 | 28.0 |
| **Stage 3** | 2110 | 45 | 255 | 35 | 321 | 38 | 1568 | 39 | 77 | 37.2 |
| **Stage 4** | 1979 | 42 | 177 | 24 | 208 | 25 | 1348 | 33.5 | 72 | 34.8 |
| **Histology** |  | | | | | | | | | |
| **NScHL** | 2936 | 63 | 577 | 78 | 591 | 72 | 2986 | 74.2 | 100 | 48.3 |
| **MScHL** | 1202 | 26 | 53 | 7 | 89 | 11 | 521 | 13 | 66 | 31.9 |
| **LRcHL** | 0 | 0 | 9 | 1 | 14 | 2 | 102 | 2.5 | 3 | 1.4 |
| **LDcHL** | 124 | 3 | 12 | 2 | 3 | 0.4 | 46 | 1.1 | 7 | 3.4 |
| **cHL, NOS** | 268 | 6 | 69 | 9 | 105 | 13 | 367 | 9.1 | 31 | 15.0 |
| **NLPHL** | 162 | 3 | 20 | 3 | 16 | 2 | 0 | 0 | 0 | 0 |
| **Other** | 0 | 0 | 0 | 0 | 5 | 0.6 | 0 | 0 | 0 | 0 |
| **B symptoms** | 3274 | 71 | 477 | 64 | 481 | 57 | 2938 | 73.1 | 161 | 77.8 |
| **Bulky disease** | 768 | 22 | 288 | 39 | NA | NA | 1408 | 35.0 | 36 | 17.4 |
| **Male sex** | 2882 | 61 | 403 | 54 | 444 | 52 | 2194 | 54.5 | 120 | 58 |
| **IPS-7 score** |  | | | | | | | | | |
| **0** | 115 | 7 | 57 | 8 | 78 | 9 | 295 | 7.3 | 9 | 4.3 |
| **1** | 360 | 22 | 195 | 26 | 214 | 25 | 944 | 23.5 | 34 | 16.4 |
| **2** | 464 | 29 | 195 | 26 | 281 | 33 | 1281 | 31.9 | 57 | 27.5 |
| **3** | 378 | 23 | 155 | 21 | 159 | 19 | 929 | 23.1 | 62 | 30.0 |
| **4** | 190 | 12 | 88 | 12 | 74 | 9 | 405 | 10.1 | 31 | 15.0 |
| **≥5** | 111 | 7 | 50 | 7 | 48 | 6 | 168 | 4.2 | 14 | 6.7 |
| **Median age, years (range)** | NA (15-65) | | 32 (16-85) | | 33 (16-83) | | 33 (18-65) | | 37 (18-82) | |
| **Chemotherapy regimens** | Doxorubicin based standard regimens (ABVD); 20% MOPP or similar regimen | | ABVD or ABVD equivalent regimen | | ABVD or Stanford V | | ABVD; Stanford V; MOPPEBVCAD; BEACOPP; escBEACOPP; BEACOPP-R; COPPEBVCAD; AVD; IGEV; HSCT | | ABVD (98%); AVD; AVD+BV; BEACOPP; CHOP; HSCT | |
| **Follow-up time** | Before 1992 | | 1980-2010 | | 1996-2006 | | 1996-2014 | | 2005-2018 | |

**Supplementary Figure S1.** Kaplan-Meier Plots of PFS.


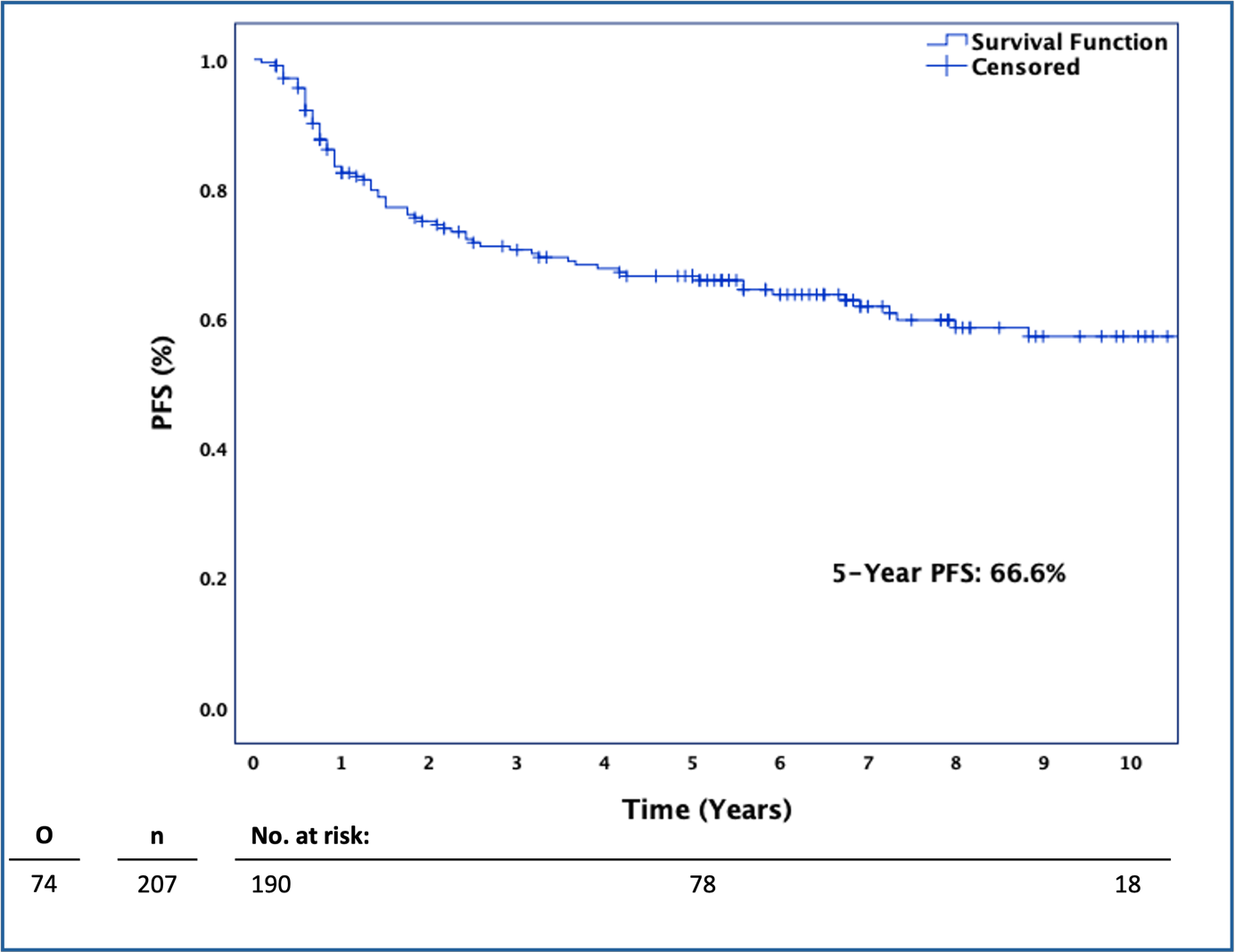


**Supplementary Figure S2.** Kaplan-Meier Plots of OS.


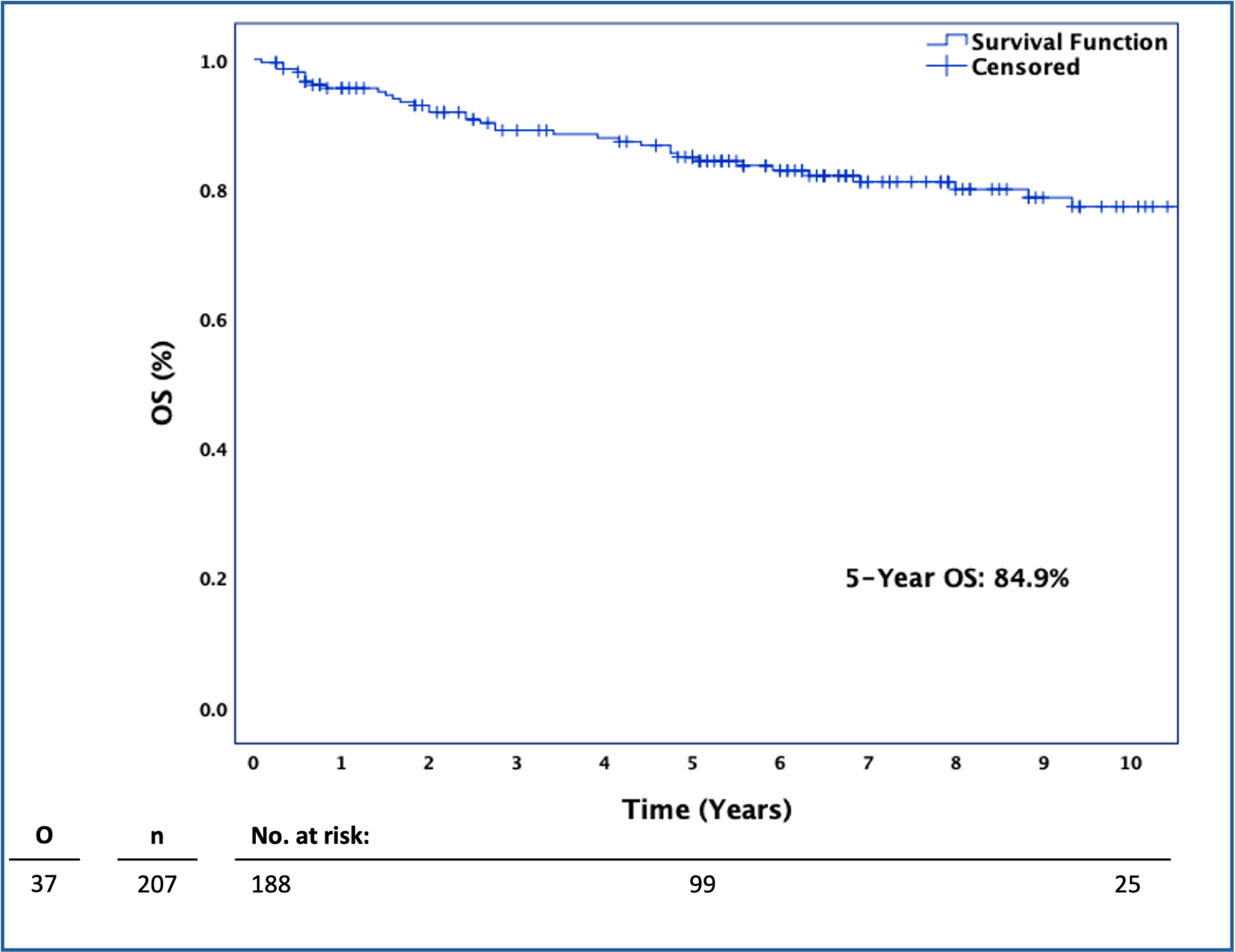

Supplement: Supplementary file 1 — Supplementary Material 1 [file 277_2025_6292_MOESM1_ESM.docx]
